# Supplementary material for: Identification and functional analysis of non-coding regulatory small RNA FenSr3 in Bacillus amyloliquefaciens LPB-18
Source: PeerJ. 2023 May 15;11:e15236. doi: 10.7717/peerj.15236 (PMC10194069; doi:10.7717/peerj.15236)
Supplement: Supplemental Information 4 [file peerj-11-15236-s004.zip › KO/CK-vs-T1_map/map03450.html]

KEGG PATHWAY: Non-homologous end-joining - Reference pathway


|  |  |
| --- | --- |
| **Non-homologous end-joining - Reference pathway** |  |

[
Pathway menu
| Organism menu
| Pathway entry
| Show description
| User data mapping
]

Nonhomologous end joining (NHEJ) eliminates DNA double-strand breaks (DSBs) by direct ligation. NHEJ involves binding of the KU heterodimer to double-stranded DNA ends, recruitment of DNA-PKcs (MRX complex in yeast), processing of ends, and recruitment of the DNA ligase IV (LIG4)-XRCC4 complex, which brings about ligation. A recent study shows that bacteria accomplish NHEJ using just two proteins (Ku and DNA ligase), whereas eukaryotes require many factors. NHEJ repairs DSBs at all stages of the cell cycle, bringing about the ligation of two DNA DSBs without the need for sequence homology, and so is error-prone.

|  |  |  |
| --- | --- | --- |
| Reference pathway | 184% 150% 122% 100% 82% 67% 55% | 图片下载 |
